# Biostimulation and microbial community profiling reveal insights on RDX transformation in groundwater

**DOI:** 10.1002/mbo3.423

**Published:** 2016-11-17

**Authors:** Dongping Wang, Hakim Boukhalfa, Oana Marina, Doug S. Ware, Tim J. Goering, Fengjie Sun, Hajnalka E. Daligault, Chien‐Chi Lo, Momchilo Vuyisich, Shawn R. Starkenburg

**Affiliations:** ^1^ Earth Systems Observations EES‐14Earth and Environmental Sciences DivisionLos Alamos National LaboratoryLos AlamosNMUSA; ^2^ Environmental Programs ADEPLos Alamos National LaboratoryLos AlamosNMUSA; ^3^ School of Science and TechnologyGeorgia Gwinnett CollegeLawrencevilleGAUSA; ^4^ Bioenergy and Biome SciencesBiology Sciences DivisionLos Alamos National LaboratoryLos AlamosNMUSA

**Keywords:** biodegradation, bioremediation, microbial structure, pseudomonas, water

## Abstract

Hexahydro‐1,3,5‐trinitro‐1,3,5‐triazine (RDX) is a high explosive released to the environment as a result of weapons manufacturing and testing worldwide. At Los Alamos National Laboratory, the Technical Area (TA) 16 260 Outfall discharged high‐explosives‐bearing water from a high‐explosives‐machining facility to Cañon de Valle during 1951 through 1996. These discharges served as a primary source of high‐explosives and inorganic‐element contamination in the area. Data indicate that springs, surface water, alluvial groundwater, and perched‐intermediate groundwater contain explosive compounds, including RDX (hexahydro‐1,3,5‐trinitro‐1,3,5‐triazine); HMX (octahydro‐1,3,5,7‐tetranitro‐1,3,5,7‐tetrazocine); and TNT (2,4,6‐trinitrotoluene). RDX has been detected in the regional aquifer in several wells, and a corrective measures evaluation is planned to identify remedial alternatives to protect the regional aquifer. Perched‐intermediate groundwater at Technical Area 16 is present at depths from 650 ft to 1200 ft bgs. In this study, we examined the microbial diversity in a monitoring well completed in perched‐intermediate groundwater contaminated by RDX, and examined the response of the microbial population to biostimulation under varying geochemical conditions. Results show that the groundwater microbiome was dominated by *Actinobacteria* and *Proteobacteria*. A total of 1,605 operational taxonomic units (OTUs) in 96 bacterial genera were identified. *Rhodococcus* was the most abundant genus (30.6%) and a total of 46 OTUs were annotated as *Rhodococcus*. One OTU comprising 25.2% of total sequences was closely related to a RDX ‐degrading strain *R. erythropolis* HS4. A less abundant OTU from the *Pseudomonas* family closely related to RDX‐degrading strain *P. putida* II‐B was also present. Biostimulation significantly enriched *Proteobacteria* but decreased/eliminated the population of *Actinobacteria*. Consistent with RDX degradation, the OTU closely related to the RDX‐degrading *P. putida* strain II‐B was specifically enriched in the RDX‐degrading samples. Analysis of the accumulation of RDX‐degradation products reveals that during active RDX degradation, there is a transient increase in the concentration of the degradation products MNX, DNX, TNX, and NDAB. The accumulation of these degradation products suggests that RDX is degraded via sequential reduction of the nitro functional groups followed by abiotic ring‐cleavage. The results suggest that strict anaerobic conditions are needed to stimulate RDX degradation under the TA‐16 site‐specific conditions.

## 1 Introduction

**Figure 1:**
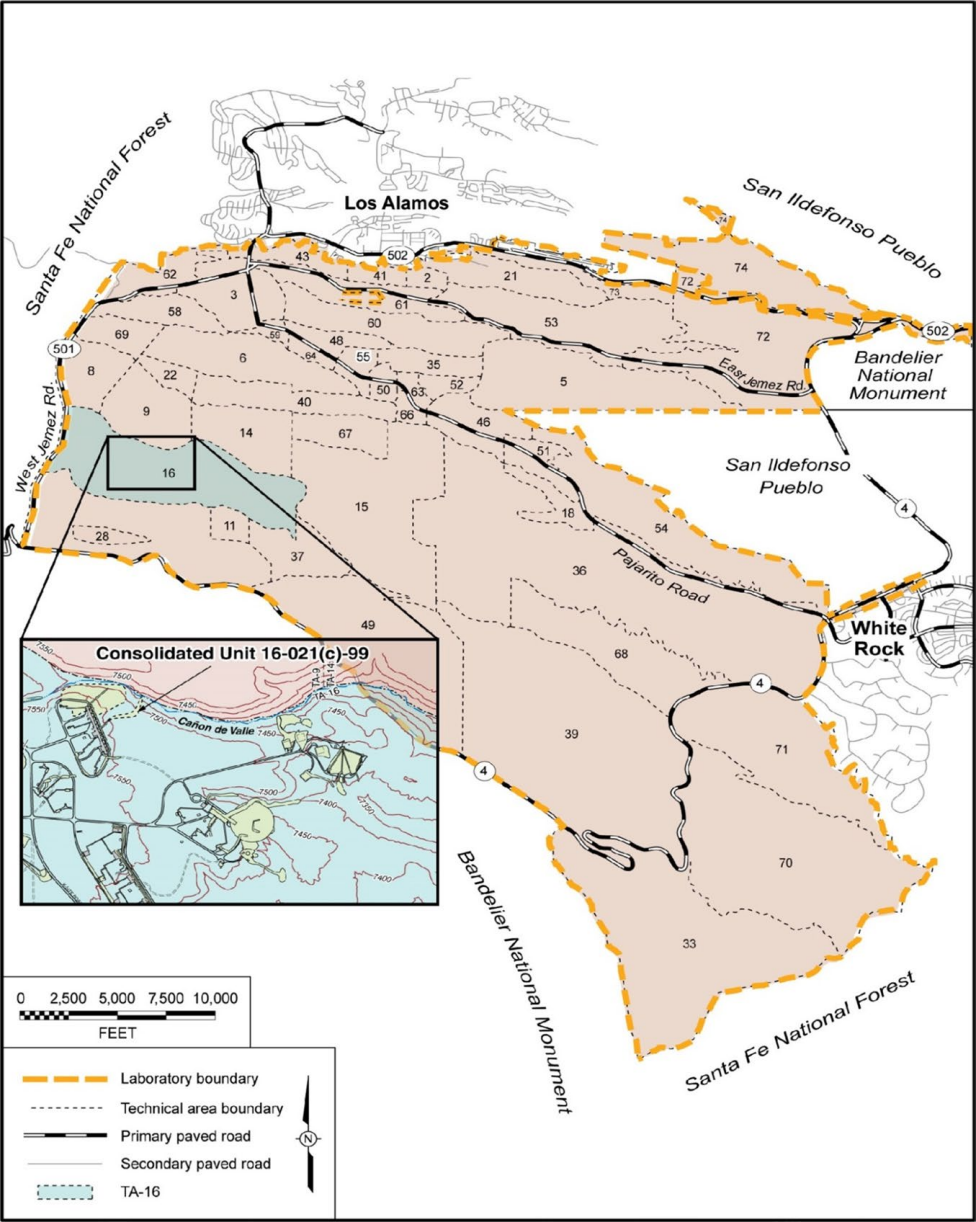
Location of TA‐16 and other Laboratory technical areas at Los Alamos National Laboratory

Hexahydro‐1,3,5‐trinitro‐1,3,5‐triazine (RDX) was widely used in explosives formulations at Los Alamos National Laboratory (LANL), Los Alamos, New Mexico. Inadequate waste water management resulted in the release of an estimated 1464 kg to 2644 kg of RDX to the TA‐16‐260 outfall in Cañon de Valle in Los Alamos (LANL (Los Alamos National Laboratory), August 2006. “Investigation Report for Intermediate and Regional Groundwater, Consolidated Unit 16‐021(c)‐99). Other high explosives (HE), such as octahydro‐1,3,5,7‐tetranitro‐1,3,5,7‐tetrazocine (HMX), 1,3,5‐triamino‐2,4,6‐trinitrobenzene (TATB), and 2‐methyl‐1,3,5‐trinitrobenzene (TNT), were also released and are detected along with their degradation products in sediments and groundwater near the processing site at TA‐16 in Los Alamos (Los Alamos, 2011; Figure 1). Most surface contaminations were remediated through cleanup operations performed in the last 10–15 years (LANL, 2010, 108868). In general, alluvial monitoring wells down‐gradient of the outfall show long‐term decreases in RDX, with concentrations currently near or below the screening level of 7.02 µg/L. The RDX concentration in the deep perched‐intermediate zone underlying the upper Cañon de Valle at TA‐16 varies between 20 and about 200 μg/L (LANL, 2015). RDX is also detected at low levels in several monitoring wells completed within the regional aquifer (LANL, 2011). RDX degradation products hexahydro‐1‐nitroso‐3,5‐dinitro‐1,3,5‐triazine (MNX), hexahydro‐1,3‐dinitroso‐5‐nitro‐1,3,5‐triazine (DNX), hexahydro‐1,3,5‐trinitroso‐1,3,5‐triazine (TNX), 4‐nitro‐2,4‐diazabutanal (NDAB), and methylenedinitramine (MEDINA) are detected in groundwater, which indicates that RDX is undergoing degradation under the natural conditions of the site. The presence of these degradation products has been attributed to the activity of microorganisms capable of degrading RDX. However, the identity of the microorganisms responsible for RDX degradation in the environment remains unknown (Fuller, McClay, Higham, Hatzinger, & Steffan, 2010; Fuller, Perreault, & Hawari, 2010). Studies undertaken in the past using stable isotope labeling and high‐throughput sequencing generally point to the importance of *Pseudomonas* and *Rhodococcus* in RDX degradation (Cho et al., 2013; Andeer et al., 2013). A number of studies have also associated RDX degradation to other genera such as *Comamonas, Clostridium, Enterobacter, Morganella, Acetobacterium, Geobacter, Citrobacter, Klebsiella, Rhizobium, Burkholderia, Shewanella,* and *Providencio* (Jayamani and Cupples, 2015b; Bhushan et al., 2002; Watrous et al., 2003; Adrian & Arnett, 2004; Bhushan, Halasz, Thiboutot, Ampleman, & Hawari, 2004; Cho, Lee, & Oh, 2008; Coleman, Spain, & Duxbury, 2002; Khan, Lee, & Park, 2012; Kitts, Cunningham, & Unkefer, 1994; Zhao, Halasz, Paquet, Beaulieu, & Hawari, 2002). These studies illustrate the difficulty in attributing the RDX degradation activity to a specific type of microorganism. The use of functional gene data along with microbial diversity data is starting to improve our understanding of which genes are involved in RDX degradation and help identify the specific microbes that are driving RDX degradation (Wilson & Cupples, 2016). Among the functional genes linked to RDX degradation *diaA*,* xenA*,* xenB*,* xplA*, and *xplB,* have received the most attention (Fuller, McClay, Hawari, Paquet, & Malone, 2009; Li et al., 2014; Wilson & Cupples, 2016).

**Scheme 1:**
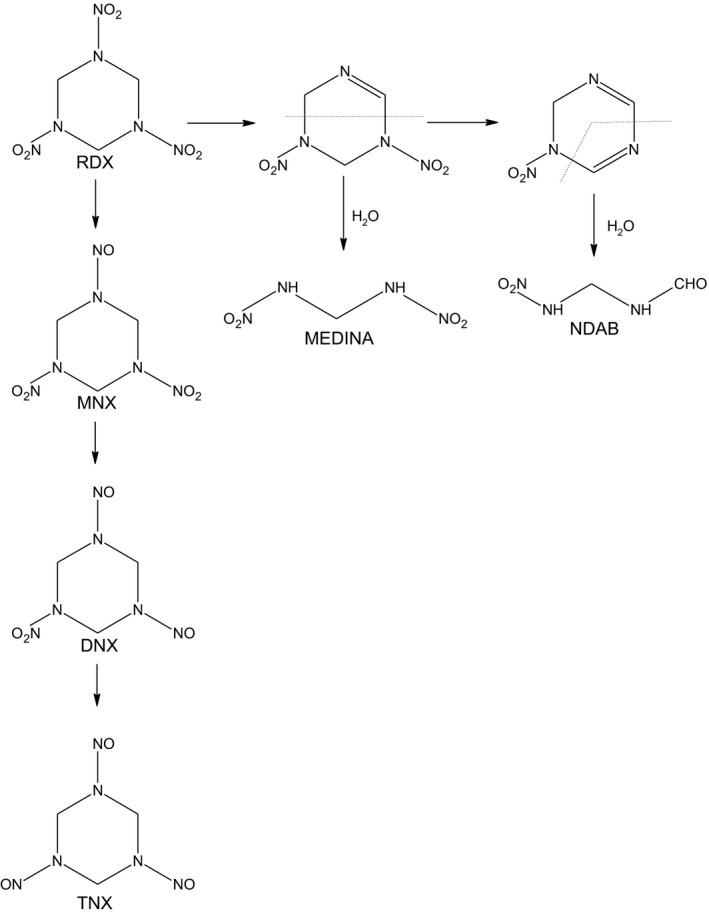
Degradation routes of RDX. Production of different transformation products depends on both biotic and abiotic factors

The degradation of RDX through anaerobic biodegradation has been extensively investigated (Scheme 1) (Beller, 2002; Bernstein & Ronen, 2012; Fournier, Halasz, Spain, Fiurasek, & Hawari, 2002; Hawari et al., 2000; Jackson, Raylot, Fournier, Hawari, & Bruce, 2007). The main degradation pathways described involve either a sequential biotic reduction of the nitro functional groups followed by abiotic ring‐cleavage (Hawari et al., 2002) or a direct denitration followed by hydration and subsequent ring‐cleavage (Jackson et al., 2007). The intermediates that accumulate in solution as a result of the sequential biotic reduction pathway include MNX, DNX, TNX, NDAB, and MEDINA. In contrast, when the breakdown pathway involves denitration and ring‐cleavage, the breakdown products include MEDINA, NDAB, nitrate, and formaldehyde. Stepwise denitration of RDX involves a nitrate reductase which is a ubiquitous enzyme possessed by a diverse group of bacteria, especially denitrifying bacteria (Bhushan et al., 2002). Degradation of RDX through denitration and ring‐cleavage involves the microbial P450 system which was shown to be able to degrade RDX under both aerobic and anaerobic conditions (Jackson et al., 2007). The cytochrome P450 system (XplA and XplB) was originally cloned from *Rhodococcus rhodochrous* (Rylott, Jackson, Sabbadin, Seth‐Smith, & Edwards, 2011; Rylott, Budarina, et al., 2011; Seth‐Smith, Rosser, Basran, Travis, & Dabbs, 2002). Expression of *xplA* and *xplB* is highly induced in the presence of RDX (Indest, Hancock, Jung, Eberly, & Mohn, 2013; Indest, Jung, Chen, Hancock, & Florizone, 2010). Recent studies have shown that production of *xplA* in Arabidopsis plants confers both the ability to remove RDX from liquid culture and resistance to the toxic effects of RDX (Rylott et al., 2006; Rylott, Jackson, et al, 2011; Rylott, Budarina, et al., 2011). *xplA* and *xplB* exist in various genera including *Rhodococcus*,* Gordonia*, and *Williamsia* are commonly found in soil and groundwater (Halasz, Manno, Perreault, Sabbadin, & Bruce, 2012). The global distribution of RDX‐degrading bacteria containing *xplA* and *xplB* gene homologs suggests that denitration may represent a key RDX degradation pathway in nature (Andeer, Stahl, Bruce, & Strand, 2009). Besides P450 enzymes, two flavin mononucleotide‐containing oxidoreductase genes *xenA* and *xenB*, have been cloned from *Pseudomonas* (Blehert, Fox, & Chambliss, 1999). Monoculture of the *Pseudomonas* strains harboring these two enzymes demonstrated that both XenA and XenB were able to degrade RDX (Fuller et al., 2009). Interestingly, XenB exhibited a broader substrate specificity than XenA. The activities of both enzymes are significantly high when degrading RDX for anaerobic conditions compared with aerobic conditions.

Biostimulation has been examined as a remediation approach to treat HE contamination including RDX. Various nutrients including acetate and edible vegetable oil are known to promote bacterial growth and RDX degradation (Livermore, Oh, LePuil, Arnseth, & Mattes, 2013; Schaefer et al., 2007). Acetate is a widely applied carbon source which enriches Fe (III)‐reducing bacteria such as *Pseudomonas*. Multiple studies have shown that Fe (III)‐reducing bacteria degrade RDX by direct reduction or indirectly by electron shuttling (Hawari et al., 2000). On the other hand, emulsified edible oils have been successfully used to enhance biodegradation of RDX. The procedures and applications of vegetable oils for the bioremediation of RDX are applicable to numerous other biodegradable contaminants like nitrates, chlorinated solvents, and perchlorates. Biostimulation using acetate and vegetable oil is normally carried out under anaerobic conditions, when RDX can act as an electron acceptor to support microbial respiration (Beller, 2002; Bernstein & Ronen, 2012). Degradation of RDX in the presence of oxygen has also been reported and where microorganisms utilize RDX as a carbon source or a nitrogen source (Fuller, McClay, et al, 2010; Fuller, Perreault, & Hawari, 2010).

In this study, we surveyed the microbial profile of RDX‐containing groundwater to determine if microorganisms are playing any active role in RDX degradation, examined the potential existence of RDX biodegradation signatures, and evaluated the response of endogenous microbes to biostimulation. This work focused on water samples collected from a well completed in deep perched‐intermediate groundwater underlying Cañon de Valle at TA‐16 at Los Alamos National Laboratory. We also performed a microcosm experiment to examine how environmental factors such as the availability of oxygen, sediments, and alternate sources of carbon affect RDX degradation. The microbial profile of the microcosms with the most RDX‐degrading activity was also determined. Our results provide insights on microorganisms and environmental conditions that are potentially important to RDX transformation in groundwater.

## 2 Materials and Methods

### 2.1 Water samples collection and processing

The CDV‐16‐4ip monitoring well is completed in deep perched‐intermediate groundwater at Technical Area 16 (TA‐16), located in the southwest corner of Los Alamos National Laboratory. The deep perched groundwater is located in several zones of saturation at depths between approximately 650 ft and 1,200 ft below ground surface (bgs). The perched groundwater is present in a variety of geologic units, including the Cerro Toledo interval, Otowi Member, and Puye Formation. These zones are potential sources of contaminated recharge to the regional aquifer. Groundwater samples were collected from CdV‐16‐4ip, screened between 815 and 879 feet bgs. Water samples used for DNA extraction were collected after pumping the well for a minimum of 3 casing volumes. The samples were immediately stored on ice in the field and during transportation, and then kept at 4°C in the laboratory until further analysis.

### 2.2 Analytical techniques

RDX and its degradation products were analyzed on a Dionex Summit HPLC (Thermo Scientific, USA) system using the EPA method (METHOD 8330A). The HPLC was equipped with an Acclaim Explosives E1 column 25 cm × 4.6 mm E1 (Thermo Scientific, Waltham, MA). The flow rate used was 0.80 ml/min and the mobile phase composition was 52% MeOH and 48% DI Water. Absorbance detection wavelength was set at 254 nm. RDX certified standards (Ultra Scientific, North Kingstown, RI) were used for sample quantification. Degradation products TNX, DNX, and MNX were obtained from SRI International, Menlo Park, CA and were used to establish calibration curves for quantitative analysis. Major anions (SO_4_^2−^, NO_3_^−^, PO_4_^3−^, HCO_3_^−^, F^−^, and Cl^−^) in the groundwater were measured using ion chromatography (Dionex, USA). Trace metals were measured with an inductively coupled plasma mass spectrometer (ICP‐MS) (Varian 810 ICP‐MS System, California) or by atomic absorbance spectrometer (Parkin Elmer, USA).

### 2.3 Microbial profiling analysis

The groundwater samples were processed immediately after reception from the field. Processing consisted of filtering 100–500 ml of the water samples using 47 mm, 0.2‐μm pore size polycarbonate filters (Thermo Scientific) to concentrate the microbial biomass. Total DNA was extracted from bacterial cells collected on each filter membrane following the method of The UltraClean^®^ Microbial DNA Isolation kit (MO BIO). DNA extracts were used to amplify the V4 region of bacterial 16S rRNA genes using bacterial barcoded primers (515F‐806R [GTGCCAGCMGCCGCGGTAA and GGACTACHVGGGTWTCTAAT, respectively]) (Hugerth et al., 2014). Amplicons (equivalent to library fragments) were purified and size selected using AMPure XP beads, quantitated by picogreen assay, normalized, pooled, and sequenced on an Illumina MiSeq instrument. The sequencing run resulted in ~100,000 paired reads per sample with an average read length of 295 bp. Paired reads were combined to produce 290 bp sequences corresponding to the V4 region and filtered to retain those with an average quality score of greater than or equal to 30. Sequence data were processed using the QIIME software package v1.9.1 (Caporaso et al., 2010). Operational taxonomic units (OTUs) were clustered at the 96% similarity level. The most abundant sequence in each cluster was chosen as a representative. Alpha diversity analysis was performed using QIIME script (alpha_rarefaction.py). To determine how bacterial community compositions varied across samples, principal coordinate analysis (PCoA) was performed by comparing unweighted UniFrac profiles for each sample in QIIME. NCBI Blast was used to assign representative sequences to genus or species levels. All sequences obtained in this study were deposited at the NCBI Sequence Read Archive (SRA) and are available under the accession number PRJNA318785 (https://submit.ncbi.nlm.nih.gov/subs/bioproject/SUB1472271/overview).

### 2.4 Phylogenetic analysis

A comparative analysis of nucleotide sequences was performed by Basic Local Alignment Search Tool (BLAST) at the National Center for Biotechnology Information (NCBI; http://www.ncbi.nlm.nih.gov/Blast.cgi/) to obtain sequences of 16S rDNA from species closely related to *Pseudomonas* and *Rhodococcus* for phylogenetic analysis. 16S rDNA sequences were aligned using CLUSTAL X (Jeanmougin, Thompson, Gouy, Higgins, & Gibson, 1998) and adjusted manually, as necessary. The resulting data matrix was first analyzed using equally weighted maximum parsimony in PAUP* (Swofford, 2002). Maximum parsimonious trees were sought using the heuristic search strategies of PAUP*. A neighbor‐joining analysis was also performed using the uncorrected pairwise nucleotide differences (“p”) in PAUP*. The confidence level of branches was evaluated by bootstrap analysis of 10,000 replicates (Felsenstein, 1985).

### 2.5 Enumeration of bacterial population

Culturable microbial populations were enumerated by dilution plate count technique (Wang, Korban, Pusey, & Zhao, 2012; Wang et al., 2015). For dilution plating, groundwater samples were serially diluted in normal sterile saline (0.9%) and 100 μl of suspension from each dilution was plated on Luria‐Bertani (LB) agar plate in triplicate and incubated at room temperature for 8 days. Total microbial counts were determined by direct count using a Hemocytometer plate (Cambridge Instruments, Inc) according to manufacturer's protocol.

### 2.6 Biostimulation and quantification of RDX and RDX degradation products

Biostimulation experiments were performed using 200 ml sterile vials containing 100 ml groundwater from the well CdV‐16‐4ip. The RDX concentration in the CdV‐16‐4ip water is around 160 ppb. The water was spiked with RDX solution to a final concentration of 1,800 ppb. The RDX solution appeared to contain NDAB and the initial NDAB concentration is about 200 ppb. Each vial was sealed with a pair of rubber and aluminum caps. Acetate (20 mmol/L) and safflower oil (1%, v/v) were added to the sealed vial at the start of the experiment using a syringe equipped with a needle. About 100 ml air was sealed within each vial to create microoxic treatments. For strict anoxic stimulation, vials were purged with nitrogen gas for 10 min to remove oxygen. Samples were incubated in the dark at room temperature (22°C) under constant shaking at 50 rpm. Samples were prepared in triplicates for the following four conditions: AC1: CdV‐16‐4ip cultivated with acetate + initial oxygen; AC2: CdV‐16‐4ip cultivated with acetate—oxygen; OIL1: CdV‐16‐4ip cultivated with safflower oil—oxygen; OIL2: CdV‐16‐4ip cultivated with safflower oil + initial oxygen. Controls with no amendments and sterile controls were also setup in triplicates to account for RDX degradation under unstimulated biotic and abiotic conditions. For abiotic controls, the vials were autoclaved at 121°C to kill indigenous microbes present in the groundwater. Triplicate control cultures were also prepared under aerobic conditions in conical flasks and amended with acetate and safflower oil. All vials were sampled routinely every 3 to 7 days by removing a sample through the rubber stopper using a 1.0 ml syringe. The samples were analyzed for RDX degradation and production of RDX degradation products, and acetate concentration. Samples were collected from each cultivation condition after 5 weeks (i.e., AC1, AC2, OIL1, OIL2, controls) and processed for microbial profiling.

## 3 Results

### 3.1 Physical and chemical characteristics of groundwater

The groundwater samples collected in our studies were obtained from well CdV‐16‐4ip which has a screened interval between 815 and 879 ft interrogating the Puye Formation. This formation is primarily made up of poorly sorted, unconsolidated, dacitic boulders, cobbles, and gravels that are either clast‐supported or matrix‐supported. Sand, silty sand, and silt are common matrix materials (LANL, 2011). The groundwater is well oxygenated with oxygen concentrations varying from 7.5 to 8.0 mg/L, the ORP measurements varied between 200 and 275 mV, and the pH is neutral typically varying between 7.33 and 7.67. The concentrations of the major anions in one of the CdV‐16‐4ip samples were: chloride = 3.5 mg/L, fluoride = 0.1 mg/L, nitrate as nitrogen = 0.89 mg/L, sulfate = 3.5 mg/L, and Na^+^ = 9.7 mg/L. The alkalinity CO_3_+HCO_3_ = 50 mg/L and its Ca^2+^ content is 10 mg/L, Mg^2+^ = 3.1 mg/L. The total organic carbon concentration is 0.57 mg/L and total dissolved solids = 126 mg/L. The concentration of RDX is about 160 ppb and the concentrations of the degradation products TNX, MNX, and DNX are typically less than 1 ppb. Culturable cell counts as enumerated using LB agar medium were about 3.6 × 10^2^ CFU/ml. In contrast, total bacteria counted using hemocytometer was 8.7 × 10^4^ cells/ml.

### 3.2 Bacterial community analysis

**Figure 2:**
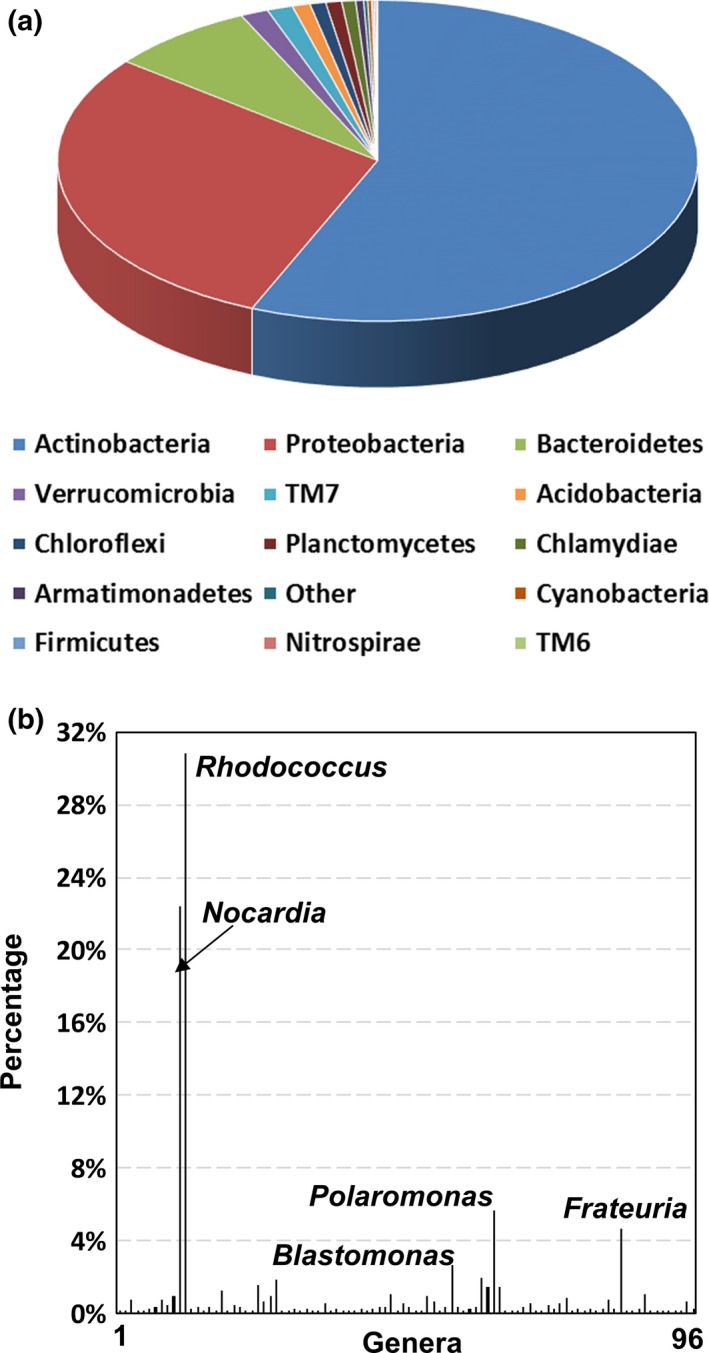
Microbial community analysis of CdV‐16‐4ip groundwater sample. (a) Relative abundances of major bacterial phyla. Data were analyzed using QIIME (Caporaso et al., 2010). (b) Relative abundances of bacterial genera in the groundwater sample. Dominant genera with ≥2% abundances were labeled near the column

A total of 98,405 bacterial 16S rRNA gene sequences were recovered for the CdV‐16‐4ip sample and used for community analyses by QIIME. OTUs were assigned by clustering sequences with over 96% sequence identity. A number of 1,605 OTUs distributed in 15 phyla were identified indicating high microbial diversity in the sample. *Actinobacteria* were dominant in the sample followed by *Proteobacteria* and *Bacteroidetes* (Figure 2a). Other members with >0.1% abundance were *Verrucomicrobia*,* Chloroflexi*,* Chlamydiae*,* Cyanobacteria*,* Armatimonadetes*,* Firmicutes*,* Planctomycetes*,* Nitrospirae*,* Acidobacteria*, TM7, TM6, and others. Identification of bacterial groups at lower taxonomic level revealed the presence of 96 genera (Figure 2b). Genera *Polaromonas*,* Frateuria*,* Blastomonas* of *Proteobacteria* and *Rhodococcus*,* Nocardia* of *Actinobacteria* represented the major populations within the communities (Figure 2b). Table S1 lists dominant OTUs (>1%) and their closest relatives found in the GenBank. The two most dominant OTUs (>20%) from CdV‐16‐4ip are identified as *Rhodococcus erythropolis* HS4 (NR_074622) with 100% sequence identity and is known to degrade RDX (Chong, 2011) and *Nocardia ignorata* DSM 44496 also with 100% sequence identity and is a human nocardiosis pathogen isolated from respiratory specimens in Europe (Rodriguez‐Nava, Couble, & Khan, 2005). Other relative abundant OTU sequences were related to (99% to 100% sequence identify) *Polaromonas jejuensis* NBRC 106434, *Frateuria aurantia* DSM 6220, *Rhodococcus cerastii* C5, *Sphingomonas desiccabilis* CP1D, *Flavobacterium macrobrachii* an‐8, *Hydrogenophaga carboriunda* YZ2, and *Pedobacter ginsengisoli* Gsoil 104.

### 3.3 *Pseudomonas and Rhodococcus*

**Figure 3:**
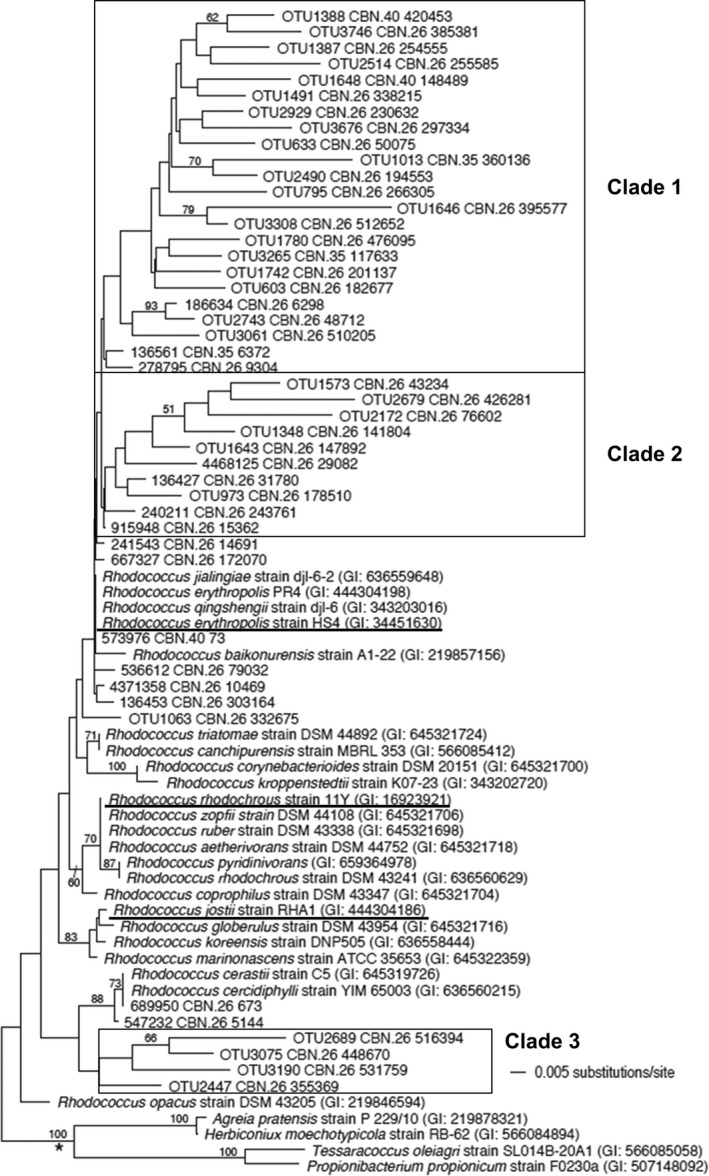
Phylogeny of *Rhodococcus* in CdV‐16‐4ip groundwater sample. Phylogenetic tree of 16S rRNA gene sequences showing the phylogenetic affiliation of the operational taxonomic units (OTUs) from the groundwater samples. The neighbor‐joining tree was constructed from the 16S rRNA V4 hypervariable sequences of representative clones of each OTU and sequences retrieved from the GenBank database. The branch indicated by “*” contains outgroups. Numbers on branches represent bootstrap estimates from 10,000 replicate analysis; values <50% are not indicated. Strain names, if any, and GenBank accession numbers are given following the species names. Known RDX‐degrading *Rhodococcus* strains were underlined

**Figure 4:**
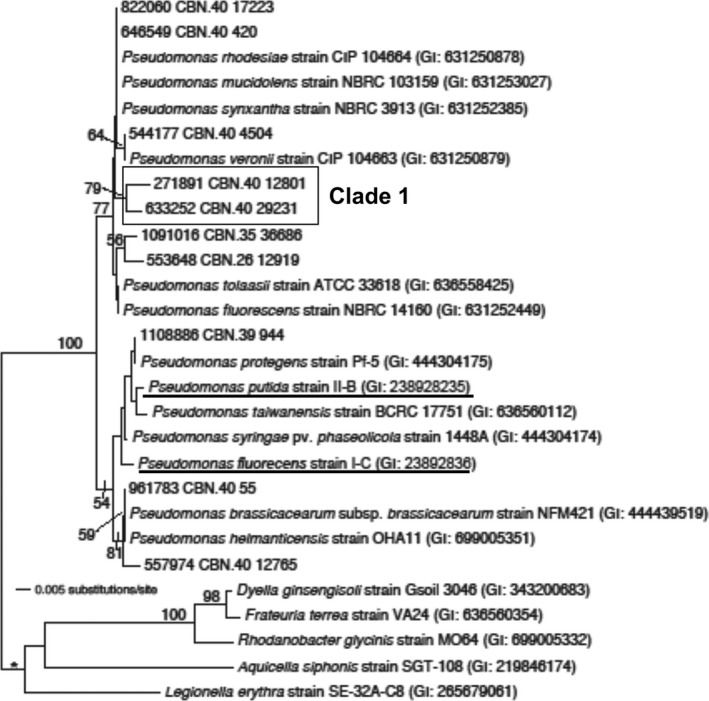
Phylogeny of *Pseudomonas* in CdV‐16‐4ip groundwater sample. Phylogenetic tree of 16S rRNA gene sequences showing the phylogenetic affiliation of the operational taxonomic units (OTUs) from the groundwater samples. The neighbor‐joining tree was constructed from the 16S rRNA V4 hypervariable sequences of representative clones of each OTU and sequences retrieved from the GenBank database. The branch indicated by “*” contains outgroups. Numbers on branches represent bootstrap estimates from 10,000 replicate analysis; values <50% are not indicated. Strain names, if any, and GenBank accession numbers are given following the species names. Known RDX‐degrading *Pseudomonas* strains were underlined

Among known RDX‐degrading genera, only *Pseudomonas* and *Rhodococcus* were detected in the CdV‐16‐4ip sample. Many *Rhodococcus* species are enriched by polycyclic aromatic hydrocarbon and related contaminants (Yu, Ke, Wong, & Tam, 2005). *Rhodococcus* was the most abundant genus in CdV‐16‐4ip (30.6%). A total of 46 OTUs were annotated as *Rhodococcus*. Phylogenetic trees of partial 16S rDNA sequences reconstructed using neighbor‐joining methods revealed evolutionary positions of the 46 OTUs in relation to 27 known *Rhodococcus* species (Figure 3). Four strains in related genera *Agreia*,* Herbiconiux*,* Tessaracoccus*, and *Propionibacterium* were selected as outgroups. All of the 46 OTUs were clustered together with *Rhodococcus* strains and separated from outgroups confirming the QIIME annotation. Based on their distance to known *Rhodococcus* species, OTUs here were grouped into two categories. One comprising those distantly related to known *Rhodococcus* strains: Clade 1 (23 OTUs), Clade 2 (10 OTUs), and Clade 3 (4 OTUs); while the other constituting 36 OTUs closely related to known species. One OTU (573976 CBN. 40 73) comprising 25.2% of total sequences was closely related to a RDX‐degrading strain *R. erythropolis* HS4 (Figure 3) (Chong, 2011). In contrast, *Pseudomonas* appeared to be less diverse and abundant than *Rhodococcus*. Most OTUs were closely clustered with reported strains except for two OTUs (boxed) in Clade 1 (Figure 4). An OTU (1108886 CBN. 39 944) was placed in the same clade with two RDX‐degrading strains *P. putida* II‐B and *P. fluorecens* I‐C (Figure 4). Together, these data suggest that the CdV‐16‐4ip groundwater harbors a diverse group of *Rhodococcus* and *Pseudomonas* including some related to RDX‐degrading strains.

### 3.4 RDX degradation in biostimulated cultures

**Figure 5:**
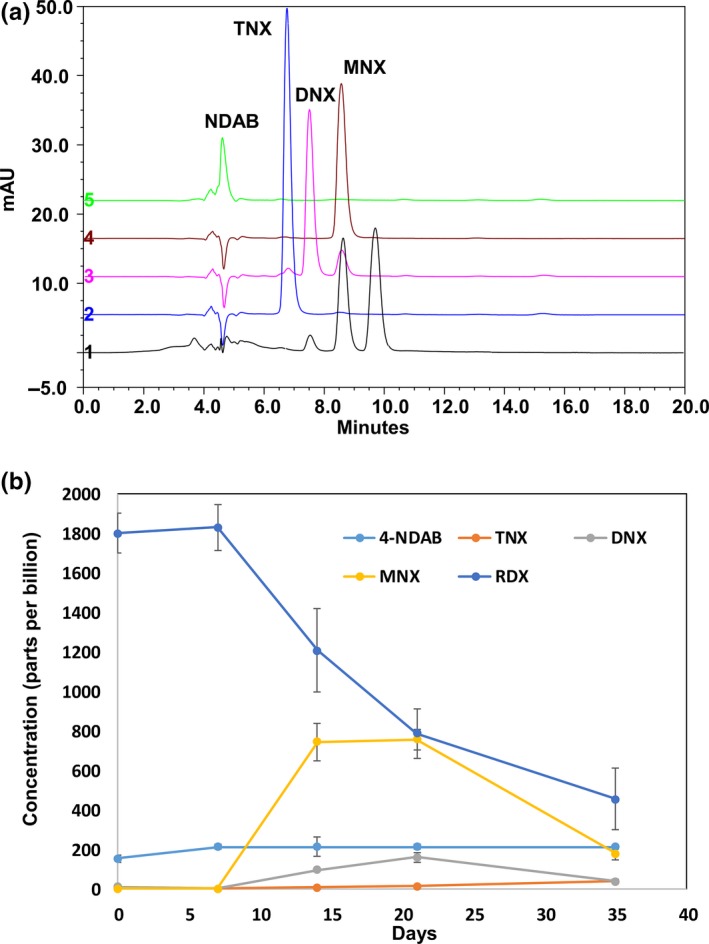
(a) HPLC chromatograms showing RDX degradation and production of RDX derivatives. 1. CDV‐16‐4ip with 2 ppm RDX was amended with 1% (v/v) safflower oil. The sample was incubated at anaerobic condition for 3 weeks. Samples were filtered to remove bacteria and other particles before HPLC analysis. 2. TNX standard. 3. DNX standard. 4. MNX standard. 5. NDAB standard. (b) Kinetics of RDX degradation and production of its derivatives. CdV‐16‐4ip (RDX ~ 2 ppm) was amended with 1% (v/v) safflower oil. The concentration of RDX, MNX, DNX, TNX, and NDAB was monitored up to 5 weeks

Both acetate and vegetable oil are known to stimulate microbial activity and enhance RDX degradation. We amended groundwater samples with acetate and safflower oil and examined RDX degradation under variable geochemical conditions to determine which factors are most relevant to RDX degradation. As shown in Table 1, little bacterial growth and RDX transformation were observed in the control samples in which no amendments were added. RDX also remained unchanged in the abiotic controls (data not shown). Acetate amendment stimulated microbial growth, with a stronger effect under microoxic conditions. However, RDX concentration were not significantly reduced in these samples over 5 weeks. A similar effect was also observed for safflower oil under microoxic environment. Even though the microbes grew to densities of 7.9 × 10^9^ cells/ml, no significant RDX degradation was observed. In contrast, safflower oil enhanced bacterial growth to the same level and promoted RDX degradation under more strict anaerobic conditions. Quantitative analysis of RDX and its degradation products by HPLC (Figure 5a, b) showed a transient increase in the concentrations of DNX and MNX during the active degradation of RDX, but their concentrations decrease rapidly when RDX degradation reaches a plateau. These data are consistent with the literature data which identified DNX, MNX, and TNX as intermediate products in the anaerobic degradation of RDX (Bernstein & Ronen, 2012). It also suggests that RDX degradation proceeds by the stepwise reduction of the nitroso groups followed by ring‐cleavage and is consistent with the mechanisms reported in the literature (Hawari et al., 2002).

**Table 1:** Effect of acetate, safflower oil, and oxygen on microbial growth and RDX degradation. Initial groundwater bacterial population was 8.7 × 10^4^ cells/ml. Final cell numbers and RDX concentrations were measured after 5 weeks

Sample ID	Treatment	Oxygen content	Bacterial cell counts after 5 weeks (cells/ml)	Culturable bacteria (cfu/ml)	Initial RDX concentration (ppm)	Final RDX concentration (ppm)
CK1	No amendments	Anoxic	1.52 × 10^5^	2.02 × 10^2^	2.58 ± 0.81	2.60 ± 0.35
CK2	No amendments	Microoxic	2.13 × 10^5^	1.46 × 10^2^	2.37 ± 0.28	2.29 ± 0.71
AC1	Acetate added	Anoxic	1.22 × 10^7^	5.19 × 10^3^	2.66 ± 0.72	2.08 ± 0.39
AC2	Acetate added	Microoxic	5.21 × 10^9^	4.81 × 10^4^	2.24 ± 0.25	1.91 ± 0.31
OIL1	Safflower oil added	Anoxic	5.83 × 10^9^	3.90 × 10^4^	2.09 ± 0.23	0.45 ± 0.34
OIL2	Safflower oil added	Microoxic	7.62 × 10^9^	5.17 × 10^5^	1.97 ± 0.15	2.24 ± 0.31

The concentration of 4‐NDAB remained fairly constant throughout the experiment and TNX was barely detectible until the 5th week. The concentration of NDAB was elevated in all samples including controls with no noticeable RDX degradation and did not change over time (Fig. S2). This is likely due to the lack of separation of NDAB from other small molecular weight metabolites present in the media which are not well resolved by the HPLC method. These results show that RDX degradation is enhanced under strict anaerobic conditions, but very negligible in the presence of oxygen. The availability of oxygen seems to inhibit RDX degradation which indicates that RDX under our current experimental conditions is degraded mainly through anaerobic respiration, which is also supported by the detection of degradation products identified as intermediates of RDX anaerobic respiration. RDX remained stable in the anaerobic reactors amended with acetate under both anoxic and microoxic conditions, although the cell numbers were significantly increased. This means that the type of nutrients provided is critical for RDX degradation possibly by differentially regulating the assemblage of groundwater microbial community.

### 3.5 Evolution of the microbial population after biostimulation

**Figure 6:**
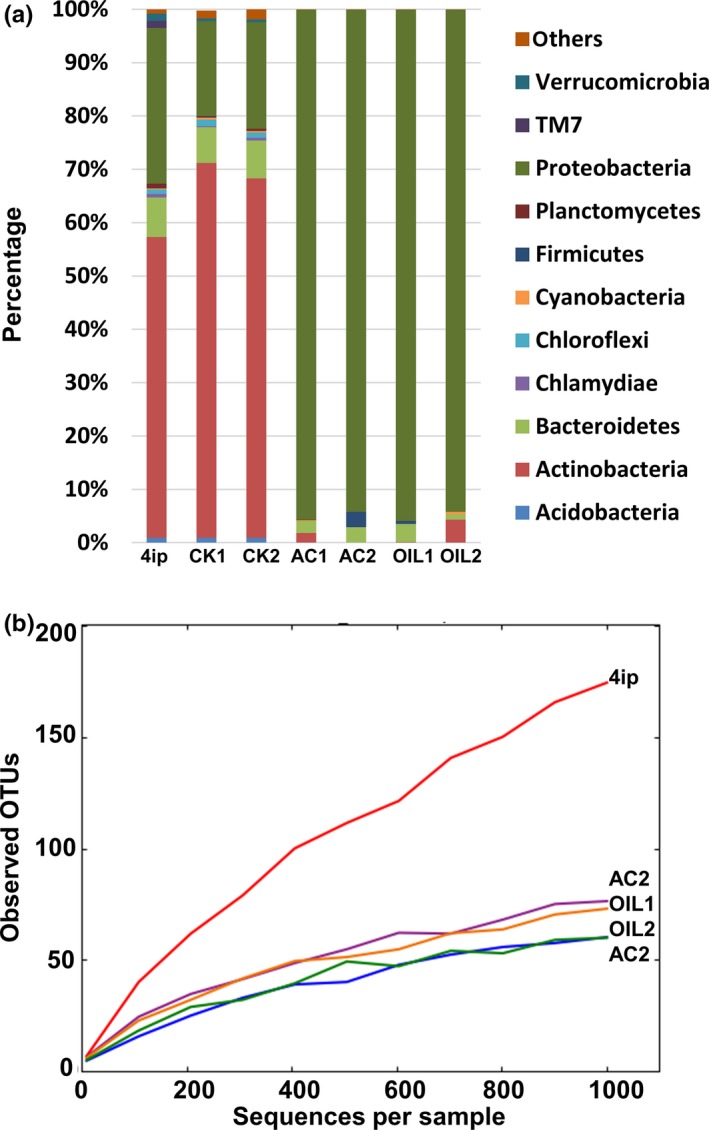
(a) Relative abundances of bacterial phyla in CdV‐16‐4ip before and after cultivation. Phyla with over 1% relative abundance were plotted. (b) Rarefaction curves for microbial diversity generated by QIIME (Caporaso et al., 2010). 200–1000 16S rRNA sequences were randomly selected and operational taxonomic units (OTUs) were calculated at a 97% cutoff. 4ip: CdV‐16‐4ip before cultivation; CK1: No amendments—oxygen; CK2: No amendments + oxygen; AC1: CdV‐16‐4ip cultivated with acetate + oxygen; AC2: CdV‐16‐4ip cultivated with acetate—oxygen; OIL1: CdV‐16‐4ip cultivated with safflower oil—oxygen; OIL2: CdV‐16‐4ip cultivated with safflower oil + oxygen

**Figure 7:**
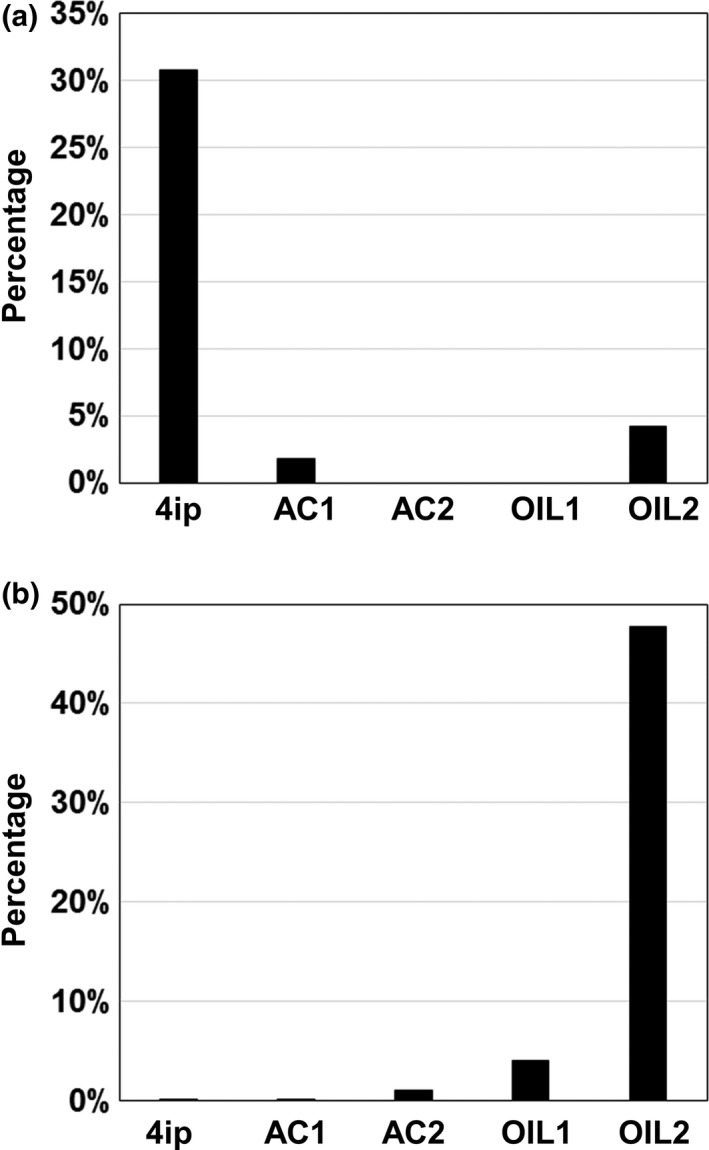
Relative abundances of *Rhodococcus* (a) and *Pseudomonas* (b) in the groundwater samples before and after cultivation. Figures were generated using numbers from QIIME analysis (Caporaso et al., 2010). 4ip: CdV‐16‐4ip before cultivation; AC1: CdV‐16‐4ip cultivated with acetate + oxygen; AC2: CdV‐16‐4ip cultivated with acetate—oxygen; OIL1: CdV‐16‐4ip cultivated with safflower oil—oxygen; OIL2: CdV‐16‐4ip cultivated with safflower oil + oxygen

To determine how nutrients and oxygen content affect the microbial community in the stimulated groundwater, we performed a microbial profiling of the bacterial communities before and 7 weeks after the biostimulation under different conditions. In the two nonstimulated control samples, a slight increase was found in *Actinobacteria* and a decrease was observed for *Proteobacteria*. In contrast, *Proteobacteria* populations were significantly increased in all stimulated samples comprising over 90% (Figure 6a). The microbes representing the phyla *Bacteroidetes*,* Nitrospirae*,* Planctomycetes*,* Chlamydiae*,* Chloroflexi,* and *Verrucomicrobia* were decreased or completely eliminated. Rarefaction curve analysis show a significant reduction of species richness after biostimulation (Figure 6b). The original 1,605 OTUs were reduced to 440, 539, 512, and 577 in the AC1, AC2, OIL1, and OIL2, respectively. Dominant OTUs representing over 67.5% of the total population were all significantly decreased, with a stronger effect under anaerobic conditions (Table 2). Three OTUs related to *Frateuria, Hydrogenophaga,* and *Pedobacter* were completely eliminated suggesting that biostimulation is detrimental to these species. *Rhodococcus* are well known as aerobic bacterial species and therefore are not able to survive under anoxic conditions. As expected, they were barely detected under anaerobic growing conditions for both acetate and vegetable oil (Figure 7a). Species in *Pseudomonas* are capable of using a range of nutrients including vegetable oil (Song, Jeon, Choi, Yoon, & Park, 2008). Therefore, they are favored when cultivated with safflower oil, especially under microoxic conditions (Figure 7b).

**Table 2:** Decrease of dominant OTUs in CdV‐16‐4ip groundwater after cultivation

OTU ID	Related bacterial strain	CdV‐16‐4ip	AC1	AC2	OIL1	OIL2
573976	*Rhodococcus erythropolis* HS4	25.20%	1.59%	0.01%	0.00%	4.08%
40439	*Nocardia ignorata* DSM 44496	21.90%	0.00%	0.00%	0.00%	0.00%
819037	*Polaromonas jejuensis* NBRC 106434	5.50%	0.02%	0.00%	0.01%	0.01%
707290	*Frateuria aurantia* DSM 6220	4.30%	0.00%	0.00%	0.00%	0.00%
689950	*Rhodococcus cerastii* C5	4.10%	0.08%	0.00%	0.00%	0.01%
1108960	*Sphingomonas desiccabilis* CP1D	2.50%	0.14%	0.00%	0.00%	0.10%
44265	*Flavobacterium macrobrachii* an‐8	1.40%	0.01%	0.00%	0.00%	0.00%
1708706	*Hydrogenophaga carboriunda* YZ2	1.30%	0.00%	0.00%	0.00%	0.00%
4339351	*Pedobacter ginsengisoli* Gsoil 104	1.30%	0.00%	0.00%	0.00%	0.00%

OTUs, operational taxonomic units.

Since OIL1 was the only sample showing RDX degradation (Figure 5; Table 1), we were particularly interested in the bacterial OTUs enriched in this sample. Table 3 lists the top nine abundant OTUs (>1%) detected in OIL1. Interestingly, none of these OTUs were dominant in the original CDV‐16‐4ip sample and their sequence abundances were all below 1%. The OTUs exhibited 99%–100% sequence identity to six known genera (*Simplicispira, Aquabacterium, Acidovorax, Curvibacter, Sphaerotilus,* and *Hydrogenophaga)* in the family of *Comamonadaceae;* two genera *(Sediminibacterium* and *Flavobacterium) of Bacteroidetes,* and one *Pseudomonas* (OTU# 1108886 showing 99% sequence identity to the RDX‐degrading strain *P. putida* II‐B) of *Pseudomonadaceae*. The *Pseudomonas* OTU (1108886) comprising 3.81% in OIL1 population was much higher than those (<1%) found in the OIL2‐, AC1‐, and AC2‐stimulated samples. Instead of OIL2 which shares the same nutrient type with OIL1, it is AC2 that showed a more similar pattern of dominant OTUs to OIL1 (Table 3). We also performed a principal coordinate analysis (PCoA) of the OTUs in the five samples (Figure S1). PC1, PC2, and PC3 accounted for 62.97%, 23.35%, and 12.53% of total variance, respectively. The score plot PC1 clearly showed that the four stimulated samples were separated from the original CdV‐16‐4ip sample. This indicates that biostimulation significantly shifts the composition of the microbial community. The two anaerobic samples AC2 and OIL1 were grouped together; while, AC1 and OIL2 were relatively far from each other in the plot. Altogether these results suggest that nutrient type and oxygen are interconnected and both are critical in shaping the microbiome during biostimulation.

**Table 3:** OTUs enriched by safflower oil under anaerobic conditions

OTU ID	Related bacterial strain (Genbank Accession #)	CdV‐16‐4ip	AC1	AC2	OIL1	OIL2
576501	*Simplicispira limi* EMB325 (NR_043773.1)	0.01%	0.03%	35.10%	28.14%	0.03%
942852	*Aquabacterium commune* B8 (NR_024875.1)	0.02%	1.32%	16.84%	26.28%	4.92%
575562	*Acidovorax delafieldii* LMG 5943 (NR_116131.1)	0.01%	0.05%	7.01%	12.65%	0.20%
895220	*Curvibacter fontanus* AQ9 (NR_112221.1)	0.14%	0.19%	3.63%	8.05%	1.71%
238109	*Sphaerotilus hippei* 566 (NR_117539)	0.65%	0.12%	0.77%	3.84%	0.32%
1108886	*Pseudomonas putida II‐B* (EF219419)	0.00%	0.05%	0.46%	3.81%	0.86%
647775	*Hydrogenophaga caeni* EMB71 (NR_043769)	0.39%	3.70%	0.94%	2.52%	11.39%
781203	*Sediminibacterium salmoneum* NJ‐44 (NR_044197)	0.02%	0.28%	0.72%	1.40%	0.38%
662742	*Flavobacterium aquaticum* JC164 (NR_108893)	0.00%	0.04%	0.51%	1.39%	0.01%

OTUs, operational taxonomic units.

## 4 Discussion

RDX degradation in groundwater is considered to be an outcome of complex interplay between the physicochemical factors and microbial activities. Although culture‐independent studies have revealed composition of bacterial communities at many RDX‐contaminated aquifers, this study provided a better understanding of community composition and environmental factors relevant for RDX transformation in groundwater at the Los Alamos site. Physicochemical analyses of water samples were performed to better understand the link between bacterial community composition and function. Our analyses showed that the groundwater samples represented the characteristic nature of nonreducing conditions, abundant HCO_3_^−^, and lack of sufficient soluble carbon nutrients. These conditions are not optimal for vigorous microbial activity that would be conductive to RDX degradation either for anaerobic respiration or utilization of RDX as a nitrogen source. Also, the utilization of RDX as a carbon source seems to be very limited under our experimental conditions. Overall, the geochemical conditions of the groundwater represent a nutritionally limited medium that is not very conductive to the indigenous microorganism activity. This is consistent with the low RDX degradation activity via microbes.

Using culture‐independent studies, we identified the presence of 96 genera in 15 phyla within the groundwater sample. A high abundance of OTUs was closely related to *Rhodococcus* strain HS4 (Figure 3) which exhibit low RDX biodegradation activities (Seth‐Smith et al., 2008). In contrast, no OTUs are closely related to *Rhodococcus* strain 11Y (Figure 3) which contain the highly effective RDX‐degrading XplA‐B system (Seth‐Smith et al., 2002). It seems that OTUs related to *Rhodococcus* strain HS4 are enriched by RDX at the Los Alamos site, whereas *Rhodococcus* 11Y‐related strains are not present. This explains the low RDX degradation activities as indicated by little RDX degradation products in the groundwater. Our study also establishes that in spite of poor nutrient content in groundwater, the bacterial diversity in the RDX‐contaminated groundwater remains significantly high.

Biostimulation has a profound impact on the microbial community. The portions of dominant OTUs were all significantly dropped after nutrient amendments (Table 2). *Polaromonas, Nocardia,* and *Rhodococcus* are known to use hydrocarbons; *Frateuria, Pedobacter,* and *Sphingomonas* include plant beneficial strains that help uptake potassium, nucleotide, and nitrogen (Subhashini, 2015; Ten et al., 2006; Videira, De Araujo, Rodrigues, Baldani, & Baldani, 2009); *Flavobacterium* and *Hydrogenophaga* are organisms that can utilize lactose and hydrogen, respectively (McCammon et al., 1998; Reinauer et al., 2014). However, none of these groups are reported to effectively utilize acetate and vegetable oil. Their suppression in the biostimulated reactors suggests their inability to grow effectively on the amendments used in this study. On the other hand, bacteria in the family of *Comamonadaceae* (*Simplicispira, Aquabacterium, Acidovorax, Curvibacter, Sphaerotilus, Hydrogenophaga*) and *Pseudomonadaceae* (*Pseudomonas*), which are capable of utilizing acetate and vegetable oil (Tang, Wu, Watson, & Parker, 2013), thrived and became dominant. Therefore, the shift in the microbial composition is reflective of the differential metabolic properties of the different microbial families. Moreover, bacteria in the families of *Pseudomonadaceae,* which are well known to synthesize various antimicrobial compounds such as antibiotics and bacteriocins (Loper et al., 2012), might have played a role in interspecies competition, and therefore a decrease in microbial diversity. This is supported by recent studies which have demonstrated that they possess type VI secretion systems involved in interspecies competitions (Decoin et al., 2014).

Acetate and vegetable oil were used to successfully stimulate RDX degradation under anaerobic conditions in different studies (Borden et al., 2004; Livermore et al., 2013). Biostimulation at field scale of an RDX‐contaminated area performed by adding acetate as a biostimulant showed a microbial population shift from a microbial community dominated by *Betaproteobacteria* to a community dominated by *Deltaproteobacteria* and *Bacteroidetes* (Livermore et al., 2013). In our study, the dominant *Actinobacteria* in CdV‐16‐4ip groundwater shifted to *Proteobacteria* (mostly *Betaproteobacteria*) under both anaerobic and microoxic conditions. This is likely reflective of the importance of the initial biogeochemical conditions of the site, especially the availability of oxygen and shows that amendments regulation of the microbiome assemblage in groundwater are likely to change significantly among different sites.

Amendment of CdV‐16‐4ip groundwater with safflower oil showed that *Gammaproteobacteria* (*Pseudomonas* in particular) were enriched in the presence of oxygen. Surprisingly, no RDX degradation is observed under aerobic conditions even under vigorous microbial activity. This is likely reflective of the inability of the microbes stimulated to utilize RDX as either a carbon or a nitrogen source or that there are abundant nitrogen and carbon in the media which are easier to utilize than RDX. Under anaerobic condition, one OTU related to RDX‐degrading *Pseudomonas spp*. was enriched by safflower oil and RDX was transformed into three RDX nitroso derivatives and NDAB (Table 3). *Pseudomonas* are known to harbor XenA and XenB which convert RDX to MNX, DNX, and TNX (Hawari et al., 2002). Although denitration and ring‐cleavage via *Rhodococcus* genes *xplA* and *xplB* generate MEDINA, NDAB, nitrate, and formaldehyde, production of the nitroso‐derivatives suggests that RDX is degraded via XenA and XenB sequential reduction by the enriched *Pseudomonas* OTU. Since the complete set of microbial species involved in RDX degradation is not well defined, it is equally possible that other bacterial genera enriched by safflower oil rather than acetate are involved in RDX degradation in anaerobic conditions. Ongoing work is being carried out to identify genes expressed under different conditions to help identify the specific microbes involved in RDX degradation.

Our microbial profiling shows that bacterial communities in the perched‐intermediate groundwater environment at TA‐16 are composed of diverse taxonomic groups. Furthermore, biostimulation analysis identified environmental conditions favoring RDX degradation. These data show that strict anaerobic conditions are required to drive biotic RDX degradation. Such conditions were also seen in remediation of contaminated aquifers that contain uranium or other radionuclides (through coffinization, etc.), which favors the immobilization of uranium (Guo et al., 2014, 2015). Our studies also show the importance of the initial availability of oxygen on the success of a bioremediation approach. Studies in an open system with continued flow are currently being carried out to specifically examine the effect of oxygen availability on microbial diversity and RDX degradation. The results will be reported in a separate paper.

## Conflict of Interest

The authors declare that they have no conflict of interest.
