# Supplementary material for: Biostimulation and microbial community profiling reveal insights on RDX transformation in groundwater
Source: Microbiologyopen. 2016 Nov 17;6(2):e00423. doi: 10.1002/mbo3.423 (PMC5387309; doi:10.1002/mbo3.423)
Supplement: Supplementary file 1 [file MBO3-6-na-s001.pdf]

Figure S1. Three-dimensional Principal Coordinates Analysis (PCoA) representation based on 16S sequencing data. 4ip: CdV-16-4ip before cultivation; AC1: CdV-16-4ip cultivated with acetate + oxygen; AC2: CdV-16-4ip cultivated with acetate - oxygen; OIL1: CdV-16-4ip cultivated with safflower oil - oxygen; OIL2: CdV-16-4ip cultivated with safflower oil + oxygen.

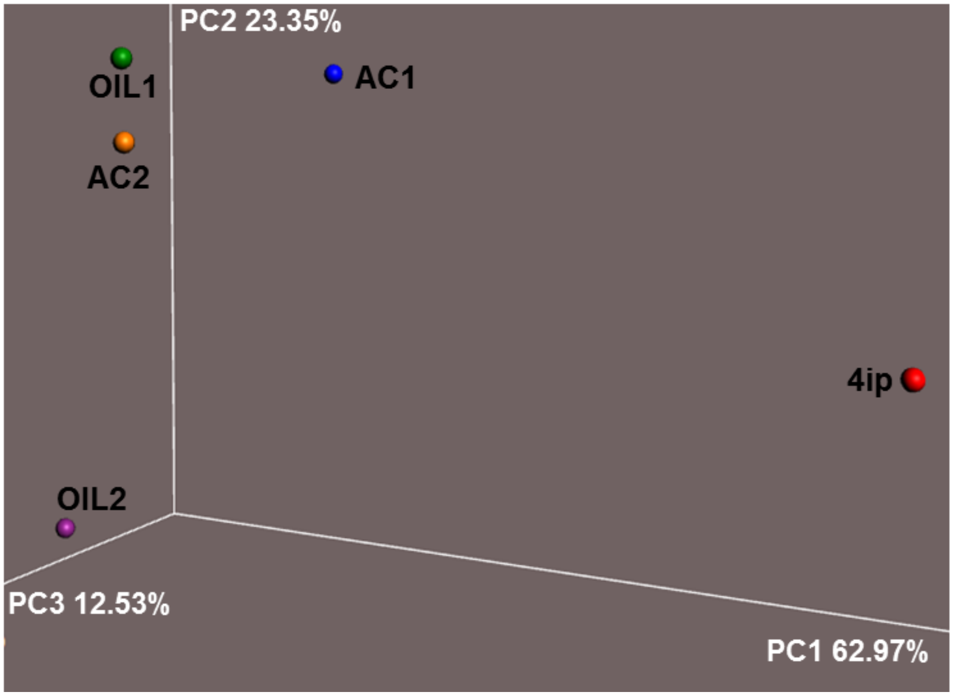

Figure S2. Plot showing the ratio of the degradation products to RDX in a control with no noticeable RDX degradation.

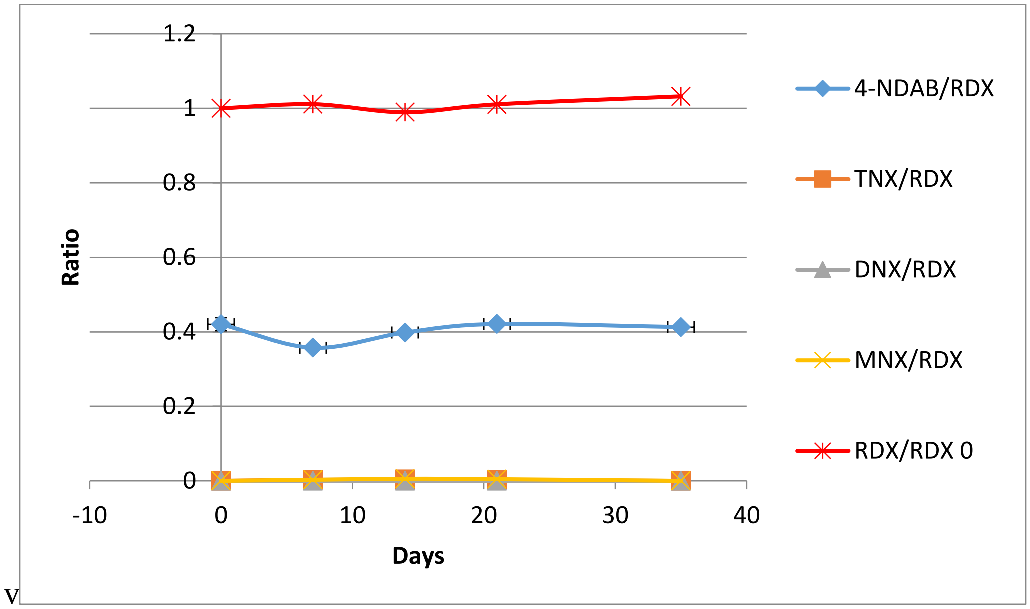

v
