# Supplementary material for: Biostimulation and microbial community profiling reveal insights on RDX transformation in groundwater
Source: Microbiologyopen. 2016 Nov 17;6(2):e00423. doi: 10.1002/mbo3.423 (PMC5387309; doi:10.1002/mbo3.423)
Supplement: Supplementary file 2 [file MBO3-6-na-s002.docx]

Table S1. Sequence identity of the dominant OTUs (>1%) obtained from QIIME analysis from the bacterial communities of the CdV-16-4ip groundwater sample

|  | **OTU ID** |  | **Relative abundances** | **Related bacterial strain** | **Sequence**  **identify** | **Accession No.** |
| --- | --- | --- | --- | --- | --- | --- |
|  | 573976 |  | 25.2% | *Rhodococcus erythropolis* HS4 | 100% | NR_074622 |
|  | 40439 |  | 21.9% | *Nocardia ignorata* DSM 44496 | 100% | NR_115832 |
|  | 819037 |  | 5.5% | *Polaromonas jejuensis* NBRC 106434 | 100% | NR_114301 |
|  | 707290 |  | 4.3% | *Frateuria aurantia* DSM 6220 | 99% | NR_074107 |
|  | 689950 |  | 4.1% | *Rhodococcus cerastii* C5 | 100% | NR_117103 |
|  | 1108960 |  | 2.5% | *Sphingomonas desiccabilis* CP1D | 99% | NR_042372 |
|  | 44265 |  | 1.4% | *Flavobacterium macrobrachii* an-8 | 99% | NR_108382 |
|  | 1708706 |  | 1.3% | *Hydrogenophaga carboriunda* YZ2 | 99% | NR_132726 |
|  | 4339351 |  | 1.3% | *Pedobacter ginsengisoli* Gsoil 104 | 100% | NR_041374 |
